# Antioxidant and Antimicrobial Potential of the *Bifurcaria bifurcata* Epiphytic Bacteria

**DOI:** 10.3390/md12031676

**Published:** 2014-03-24

**Authors:** André Horta, Susete Pinteus, Celso Alves, Nádia Fino, Joana Silva, Sara Fernandez, Américo Rodrigues, Rui Pedrosa

**Affiliations:** 1 Marine Resources Research Group (GIRM), School of Tourism and Maritime Technology (ESTM), Polytechnic Institute of Leiria, 2520-641 Peniche, Portugal; E-Mails: andre.horta@ipleiria.pt (A.H.); susete.pinteus@ipleiria.pt (S.P.); celso.alves@ipleiria.pt (C.A.); nadia.fino@ipleiria.pt (N.F.); joana.m.silva@ipleiria.pt (J.S.); arodrigues@ipleiria.pt (A.R.); 2 Higher School of Agricultural Engineering (ETSEA), University of Lleida, E-25003 Lleida, Spain; E-Mail: sara_lofer@hotmail.com; 3 Gulbenkian Institute of Science, 2780-156 Oeira, Portugal; 4 Center of Pharmacology and Chemical Biopathology, Faculty of Medicine, University of Porto, 4200-319 Porto, Portugal

**Keywords:** marine microorganisms, natural compounds, human pathogenic, *Alteromonas* sp., *Shewanella* sp., antibacterial, surface-associated microorganisms, marine symbiosis, macro-algae, seaweeds

## Abstract

Surface-associated marine bacteria are an interesting source of new secondary metabolites. The aim of this study was the isolation and identification of epiphytic bacteria from the marine brown alga, *Bifurcaria bifurcata*, and the evaluation of the antioxidant and antimicrobial activity of bacteria extracts. The identification of epiphytic bacteria was determined by 16S rRNA gene sequencing. Bacteria extracts were obtained with methanol and dichloromethane (1:1) extraction. The antioxidant activity of extracts was performed by quantification of total phenolic content (TPC), 2,2-diphenyl-1-picrylhydrazyl (DPPH) radical scavenging activity and oxygen radical absorbance capacity (ORAC). Antimicrobial activities were evaluated against *Escherichia coli*, *Pseudomonas aeruginosa*, *Bacillus subtilis*, *Salmonella enteritidis*, *Staphylococcus aureus*, *Saccharomyces cerevisiae* and *Candida albicans*. A total of 39 *Bifurcaria bifurcata*-associated bacteria were isolated and 33 were identified as *Vibrio* sp. (48.72%), *Alteromonas* sp. (12.82%), *Shewanella* sp. (12.26%), *Serratia* sp. (2.56%), *Citricoccus* sp. (2.56%), *Cellulophaga* sp*.* (2.56%), *Ruegeria* sp*.* (2.56%) and *Staphylococcus* sp*.* (2.56%). Six (15.38%) of the 39 bacteria *Bifurcaria bifurcata*-associated bacteria presented less than a 90% Basic Local Alignment Search Tool (BLAST) match, and some of those could be new. The highest antioxidant activity and antimicrobial activity (against *B. subtilis*) was exhibited by strain 16 (*Shewanella* sp.). Several strains also presented high antimicrobial activity against *S. aureus*, mainly belonging to *Alteromonas* sp*.* and *Vibrio* sp. There were no positive results against fungi and Gram-negative bacteria. *Bifurcaria bifurcata* epiphytic bacteria were revealed to be excellent sources of natural antioxidant and antimicrobial compounds.

## 1. Introduction

The marine environment is extremely complex and contains a huge diversity of life forms, which is a huge source of biological and chemical diversity [1,2,3]. The complex interactions between biological and chemical factors stimulate marine organisms to produce a high spectrum of different compounds with biological activities [4,5]. Among other reasons, marine organisms produce these compounds as a response to ecological pressures, since they survive and live within complex communities and in close association with other organisms in a very exigent, competitive and aggressive surrounding [4,6]. Sharing a common environment over a long evolutionary period also allowed the establishment of well-balanced associations between many of these organisms [7]. The surfaces of marine eukaryotes provide a unique habitat for microorganisms colonization, where competition between members of these communities and chemically-mediated interactions with their host are thought to influence both microbial diversity and function [8]. This close association of marine eukaryotes with microorganisms improves their physiological capacities, mainly providing microbial defenses [9]. For sessile marine organisms, like macro-algae, this association is an important strategy of survival in the environment where they live, since it is known that marine microorganisms strengthen the host defense mechanism by producing various kinds of secondary metabolites. Many of these metabolites have been recognized to be rich in chemical and biological properties [10,11]. Studies focusing on symbiotic relationships in marine environment indicate that many bioactive compounds previously found in marine animals and plants were in fact produced or metabolized by associated microorganisms [12,13,14]. For these reasons, in recent years, microorganisms associated with the surfaces of marine eukaryotes have been major targets for the discovery of new bioactive metabolites [15].

Among marine organisms, macro-algae can host varieties of heterotrophic bacteria, and many of these bacteria play an important role in maintaining the health of the host organism by producing unique bioactive secondary metabolites. With the increasing need for novel drug discovery, an understanding of the marine epiphyte-host associations outlined above is likely to deliver a rich source of preexisting and of new high value-added biomolecules with the potential for providing sustainable economic and human benefits [8,9,16,17]. These bioactive compounds with biomedical potential can have an important role in the prevention or treatment of human diseases, like infectious diseases and diseases associated with oxidative damage that have a high impact in world society.

*Bifurcaria bifurcata* is a perennial brown alga, found abundantly along the North East Atlantic shores, from Ireland as the northern limit, to south of the Western Sahara as the southern limit [18]. Previous studies reported a high antioxidant and antimicrobial potential in *Bifurcaria bifurcata* extracts [19,20]. However, little is known about the biodiversity of its associated bacteria and their capacity in the production of bioactive compounds. Therefore, the aim of this study was the isolation and identification of epiphytic bacteria from *Bifurcaria bifurcata* and the evaluation of the antioxidant and antimicrobial activities of extracts obtained from the isolated strains.

## 2. Results and Discussion

The oceans are massively complex and consist of diverse assemblages of life forms. The water column of the oceans contains approximately 10^6^ bacterial cells per milliliter [21]. In this study, 39 phenotypic different bacteria from the *Bifurcaria bifurcata* surface were isolated, and the strains were subjected to molecular identification using 16S rRNA gene sequencing.

As shown in Table 1, *Vibrio* sp. was the most common bacteria, representing 48.72% of all the *B. bifurcata* isolated bacteria. Bacteria belonging to the Vibrionaceae family are widespread in the marine environment. This family is particularly abundant on the surface of marine macroorganisms, where they form commensal, symbiotic or pathogenic associations [22]. *Alteromonas* sp. and *Shewanella* sp. were the following more representative genus, with 12.82% and 12.26% of occurrence, respectively. Both strains are frequent in the marine environment and have already been found to be associated with marine invertebrates, namely sponges and algae [23,24]. *Cellulophaga* sp., *Serratia* sp., *Ruegeria* sp., *Staphylococcus* sp. and *Citricoccus* sp. were represented by one strain each (2.56%). However, these bacteria are found to be frequently associated with marine invertebrates and have been the focus of study by other authors [11,25,26,27,28].

**Table 1:** The identification of epiphytic bacteria isolated from *Bifurcaria bifurcata*, at the genus level. BLAST, Basic Local Alignment Search Tool.

Epiphytic Bacteria	Genus	Occurrence (%)
1	*Citricoccus* sp.	2.56
2; 3; 10; 11; 13; 14; 15; 18; 20; 21; 22; 23; 29; 31; 35; 39; 40; 50; 51	*Vibrio* sp*.*	48.72
4	*Cellulophaga* sp*.*	2.56
6; 12; 28; 30; 34	*Alteromonas* sp*.*	12.82
13B	*Serratia* sp.	2.56
16; 17; 25; 26	*Shewanella* sp*.*	12.26
27	*Ruegeria* sp*.*	2.56
32	*Staphylococcus* sp*.*	2.56
8; 9; 24; 36; 38; 44	<90% BLAST match	15.38

Marine bacteria develop unique metabolic and physiological capabilities, which enable them to survive in extreme habitats and to produce compounds that might not be produced by their terrestrial counterparts [1,6].

In this work, marine bacteria were isolated from the brown alga, *Bifurcaria bifurcata*, and screened for their antioxidant and antimicrobial capacities. Since there is not a specific method to evaluate the antioxidant activity of a compound/extract, due to different antioxidant mechanisms, the antioxidant activity was evaluated through three methods, namely, oxygen radical absorbance capacity (ORAC), 2,2-diphenyl-1-picrylhydrazyl (DPPH) radical scavenging activity and total phenolic content (TPC).

The antioxidant results are stated in Table 2. The TPC was evaluated by the Folin-Ciocalteu method, and the results are expressed as gallic acid equivalents per gram of extract (GAE/g extract). Bacteria 16, belonging to the *Shewanella* genus, showed the highest phenolic content (53.854 ± 3.02 GAE/g extract) followed by Bacteria 8 (14.222 ± 4.25 GAE/g extract) and 36 (10.376 ± 1.58 GAE/g extract). There are not many works quantifying the total phenolic content of marine bacteria, especially epiphytic algae. However, a total phenolic content of 0.22 mg GAE/g of extract was found on the marine Actinobacteria, which was significantly lower when compared with the results obtained by the *B. bifurcata*-associated bacteria study [29]. Moreover, the phenolic content found on Bacteria 16 was surprisingly high, even when compared with the total phenolic content of some macro-algae [30].

**Table 2:** Antioxidant activity of *Bifurcaria bifurcata* epiphytic bacteria. The results are the mean ± SEM of four independent experiments. TPC, total phenolic content; GAE, gallic acid equivalents; DPPH, 2,2-diphenyl-1-picrylhydrazyl; ORAC, oxygen radical absorbance capacity; TE, trolox equivalents; BHT, butylated hydroxytoluene.

Epiphytic Bacteria	TPC (mg GAE/g Extract)	DPPH Radical Scavenging Activity IC_50_ (µg/mL)	ORAC (µmol TE/g Extract)	
1	3.040 ± 0.32	>1000	417.245 ± 9.44	
2	7.120 ± 0.05	>1000	43.220 ± 6.41	
3	2.080 ± 0.19	>1000	254.218 ± 6.47	
4	1.352 ± 0.09	>1000	154.985 ± 3.04	
6	2.923 ± 0.05	>1000	242.595 ± 6.68	
8	14.222 ± 4.25	164.40 (109.40–247.10)	912.621 ± 23.79	
9	4.870 ± 0.02	>1000	34.420 ± 1.63	
10	6.090 ± 0.07	>1000	20.400 ± 4.99	
11	6.530 ± 0.05	183.80 (118.50–285.20)	31.220 ± 2.06	
12	2.540 ± 0.30	>1000	215.289 ± 7.46	
13	9.750 ± 0.04	>1000	33.950 ± 4.40	
14	5.990 ± 0.06	>1000	47.950 ± 5.77	
15	5.390 ± 0.07	>1000	29.370 ± 2.62	
16	53.854 ± 3.02	20.21 (14.41–28.34)	3603.659 ± 53.38	
17	6.470 ± 0.09	>1000	44.250 ± 2.28	
18	1.633 ± 0.19	>1000	202.810 ± 4.75	
20	7.140 ± 0.01	>1000	52.110 ± 8.20	
21	1.668 ± 0.06	>1000	131.556 ± 2.36	
22	0.878 ± 0.06	>1000	119.415 ± 3.05	
23	2.035 ± 0.15	>1000	561.990 ± 12.69	
24	3.220 ± 0.25	>1000	407.490 ± 5.39	
25	9.380 ± 0.06	>1000	42.720 ± 4.08	
26	5.656 ± 0.13	521.00 (366.90–739.70)	520.917 ± 14.13	
27	2.640 ± 0.01	>1000	12.210 ± 0.68	
28	1.848 ± 0.21	>1000	149.545 ± 5.53	
29	1.117 ± 0.13	>1000	67.211 ± 0.99	
30	3.502 ± 0.12	>1000	68.979 ± 0.93	
31	8.556 ± 1.89	>1000	692.260 ± 13.61	
32	1.963 ± 0.21	>1000	201.190 ± 4.99	
34	2.220 ± 0.16	>1000	204.646 ± 1.89	
35	2.770 ± 0.04	>1000	358.226 ± 14.32	
36	10.376 ± 1.58	587.70 (442.00–781.40)	129.665 ± 0.91	
38	5.340 ± 0.07	>1,000	55.290 ± 3.11	
39	7.150 ± 0.03	23.62 (19.45–28.68)	36.100 ± 4.69	
40	5.900 ± 0.02	>1000	71.000 ± 15.49	
44	3.772 ± 0.44	>1000	97.450 ± 2.11	
50	4.106 ± 0.02	>1000	33.120 ± 0.42	
51	1.586 ± 0.89	>1000	246.910 ± 9.97	
13B	4.270 ± 0.02	>1000	18.880 ± 0.24	
BHT	-	40.55 (27.39–60.05)	-	

Regarding the DPPH radical scavenging activity, the highest potency was produced by Bacteria 16 (*Shewanella* sp.) and Bacteria 39 (*Vibrio* sp.), which showed an IC_50_ of 20.21 (14.41–28.34) µg/mL and 23.6 (19.5–28.7) µg/mL, respectively. The Bacteria 8, 11, 26 and 36 also denoted quite high antioxidant activity on the reduction of DPPH with an IC_50_ of 164 (109–247) µg/mL, 183.8 (118.5–285.2) µg/mL, 521 (366.9–739.7) µg/mL and 587.7 (442–781.4) µg/mL, respectively. These results are extremely interesting, especially when compared with the synthetic antioxidant, BHT (butylated hydroxytoluene), which presented an IC_50_ of 40.55 (27.39–60.05) µg/mL, since Bacteria 16 and 39 presented more potency in scavenging the DPPH radical. Kalirajan and co-workers [31] evaluated the DPPH radical scavenging activity of bacteria associated with a marine sponge and obtained an IC_50_ of 857.49 µg/mL, which is 40 times less potent than that obtained for Bacteria 16 and 39 in our work.

Recently, the ORAC assay has received much attention as a new *in vitro* method for measuring the antioxidant activity of marine extracts by evaluating their ability to scavenge certain peroxyl-radicals that induce oxidation in the presence of fluorescein [32]. According to Zulueta and co-workers [33], the ORAC method is the only one so far that combines the total inhibition time and the percentage of the free-radical damage by the antioxidant into a single quantity, ensuring that, by the end of the process, all the antioxidants present in the sample have reacted with the radicals generated. Further evaluation of the peroxyl scavenging activity of the *B. bifurcata* epiphytic bacteria extracts was also conducted by ORAC assay, and the results were expressed as trolox equivalents per gram of extract (TE/g extract). Once more, Bacteria 16 showed the highest antioxidant activity with an ORAC value of 3603.659 ± 53.38 µmol of TE/g extract, followed by Bacteria 8, 31, 23 and 26 with 912.621 ± 23.79 µmol of TE/g extract, 692.260 ± 13.61 µmol of TE/g extract, 561.990 ± 12.69 µmol of TE/g extract and 520.917 ± 14.13 µmol of TE/g extract, respectively. The lack of information about the production of antioxidant compounds produced by marine epiphytic bacteria, namely evaluated by the ORAC method, does not allow us to make a direct comparison with other works; however, Wang and co-workers [34] evaluated the peroxyl radical scavenging activity also by the ORAC system in 10 macro-algae species from Iceland, and they obtained values ranging from 4 to 2567 µmol of TE/g extract, which are values substantially lower than the results obtained by our bacteria extracts, since the lowest result was 12.210 ± 0.68 µmol of TE/g of extract (Bacteria 27) and the highest was 3603.659 ± 53.38 µmol of TE/g of extract (Bacteria 16).

The high antioxidant activity of *B. bifurcata* epiphytic bacteria presented in this work can be explained by the environmental conditions where these bacteria exist. They live in close association with soft-bodied marine organisms, which lack obvious structural defense mechanisms, and, thus, rely on chemical defense by the production of bioactive secondary metabolites to survive in their extreme habitat [1]. The production of ROS is prevalent in the world’s oceans, and oxidative stress is an important component of the stress response in marine organisms. In marine systems, the absorption of solar radiation by dissolved organic matter in seawater leads to the photochemical production of diverse reactive transients, including ROS. Since marine organisms cannot avoid these challenges, they produce compounds that can operate as antioxidant defenses [35].

It is well known that the genus, *Shewanella* (Bacteria 16), produces long-chain polyunsaturated fatty acids, and recent studies associated these compounds with an efficient antioxidant system mechanism, which operates as a primary protective “breakwater” for all marine microorganisms possessing them [35]. This can explain the high antioxidant activity shown by Bacteria 16 (*Shewanella* sp.).

Besides the production of antioxidant compounds, marine bacteria also have the ability to produce antimicrobial compounds that play an important role in the protection against other pathogenic and fouling microorganisms [22].

In this work, the antimicrobial activity of compounds produced by *B. bifurcata* epiphytic bacteria was also evaluated against several microorganisms, including human pathogens, namely *Staphylococcus aureus*, *Escherichia coli*, *Pseudomonas aeruginosa*, *Candida albicans*, *Saccharomyces cerevisiae*, *Salmonella enteritidis* and *Bacillus subtilis*. Extracts were tested against all the mentioned microorganisms at 1 mg/mL; the ones that presented a capacity to inhibit the microorganism’s growth in more than 50% (IC_50_ < 1 mg/mL) were also evaluated through dose-response analysis (10, 30, 100, 300 and 1000 µg/mL), and the IC_50_ was determined. The *B. bifurcata*-associated bacteria extracts did not show antimicrobial activity against *Escherichia coli*, *Pseudomonas aeruginosa*, *Candida albicans*, *Saccharomyces cerevisiae* and *Salmonella enteritidis* (data not shown). However, several of the *B. bifurcata*-associated bacteria extracts induced high antibacterial activity against *Bacillus subtilis* and *Staphylococcus aureus* (Table 3). The obtained IC_50_ ranged from 2.29 µg/mL (1.79–2.94) to 621.4 µg/mL (508.2–759.9) exhibited by Bacteria 16 (*Shewanella* sp.) and 13 (*Vibrio* sp.), respectively, against *B. subtilis*, and 50.85 µg/mL (40.72–63.50) to 722.9 µg/mL (541.11–965.9) exhibited by Bacteria 34 (*Alteromonas* sp.) and 4 (*Cellulophaga* sp.), respectively, against *S. aureus*. Actually, the potency of some bacteria extracts against *B. subtilis* growth, such as Bacteria 16 (*Shewanella* sp.), 25 (*Shewanella* sp.), 17 (*Shewanella* sp.), 34 (*Alteromonas* sp.) and 24, was even higher than that of the commercial antibiotic, chloramphenicol. Moreover, the *B. bifurcata*-associated bacteria extracts from the Bacteria 13B (*Serratia* sp.), 39 (*Vibrio* sp.), 50 (*Vibrio* sp.) and 38 had a chloramphenicol similar potency.

**Table 3:** Antibacterial activity of *Bifurcaria bifurcata* epiphytic bacteria. The results are the mean ± SEM of eight independent experiments. Bacitracin, chloramphenicol, oxytetracycline and ampicillin were used as positive controls.

Epiphytic bacteria	*B. subtilis*	*S. aureus*
	IC_50_ (µg/mL)	IC_50_ (µg/mL)
2	95.06 (82.41–109.7)	>1000
3	122.4 (87.66–170.9)	95.54 (67.26–135.7)
4	>1000	722.9 (541.11–965.9)
6	102.5 (68.34–153.9)	63.48 (52.77–76.38)
9	532.3 (452.2–626.6)	>1000
13	621.4 (508.2–759.9)	>1000
14	148.5 (115.9–190.4)	>1000
15	126.4 (91.68–174.2)	>1000
16	2.29 (1.79–2.94)	>1000
17	36.97 (29.97–45.60)	>1000
18	98.32 (84.47–114.4)	>1000
23	739 (525.5–1039)	136.6 (102.8–181.6)
24	47.39 (30.12–74.58)	>1000
25	31.01 (21.49–44.75)	>1000
30	71.35 (54.19–93.94)	>1000
32	>1000	127.1 (85.79–188.4)
34	24.17 (14.9–39.20)	50.85 (40.72–63.50)
35	303.3 (144.5–636.5)	>1000
38	58.87 (41.39–83.74)	>1000
39	58.86 (43.60–79.46)	>1000
40	411.5 (323.8–522.9)	>1000
50	62.54 (39.57–98.86)	>1000
13 B	52.42 (37.00–74.27)	>1000
Bacitracin	4.09 (3.30–5.06)	4.05 (3.35–4.90)
Chloramphenicol	48.14 (33.73–68.71)	26.01 (19.06–35.5)
Oxytetracycline	0.16 (0.128–0.19)	0.40 (0.265–0.610)
Ampicillin	0.16 (0.124–0.21)	0.04 (0.028–0.05)

The extracts from Strains 34 and 6, which belong to the *Alteromonas* genus, showed the highest antimicrobial activity against *Staphylococcus aureus* with an IC_50_ of 50.85 and 43.48 µg/mL, respectively. In line with our results, Barja and co-workers [36] isolated antimicrobial compounds from *Alteromonas* sp. that exhibited a broad inhibition, including against *Staphylococcus aureus*. Furthermore, Shiozawa and co-workers isolated a new antibiotic also produced by *Alteromonas* sp. with high antibacterial activity [37]. In another study, *Alteromonas* sp. isolated from a marine invertebrate also exhibited high antimicrobial activity, namely against fish pathogenic bacteria [38].

It is widely accepted that marine organisms produce compounds to survive and adapt to the unfavorable conditions where in which they live, and the production of antimicrobial compounds by marine bacteria seems to be a powerful weapon in space competition, namely for surfaces colonization. Other strains of *Bacillus* are current in the marine environment. For instance, *Bacillus licheniformis* (strain EI-34-6) and *Bacillus subtilis* (strain II-111-5) were isolated from the marine alga, *Palmaria palmate* [39]. Therefore, *Bacillus* strains are direct competitors of other species of marine epiphytic bacteria, like *Shewanella* sp., *Alteromonas* sp., *Serratia* sp. and *Vibrio* sp., which can explain the high antimicrobial activity of these bacteria’s extracts against *Bacillus subtilis*. In line with these findings, it was not surprising that more than 50% of the total bacteria isolated from the *B. bifurcata* macro-alga presented antibacterial activity against *B. subtilis*.

According to the literature, the production of antimicrobial compounds by marine organisms against Gram-positive bacteria seems to be more frequent [1]. The generally low activity of compounds against Gram-negative organisms may be due to the fact that Gram-negative bacteria possess an outer membrane and a periplasmic space, both of which are absent in Gram-positive bacteria. The outer membrane of Gram-negative bacteria is known to present a barrier to the penetration of numerous antibiotic molecules. In addition, the periplasmic space contains enzymes that are capable of breaking down foreign molecules introduced from the outside [40]. Moreover, microbial competition for limited natural resources within a community is thought to be an important selective force that promotes the biosynthesis of antimicrobial compounds. From an ecological point of view, the inhibition of other marine bacterial species competing for the same niche will give a selective advantage during colonization [41]. In this study, the weak inhibitory effect against most bacteria and fungi can provide evidence of a lack of competition for the same niche.

In this study, some bacterial extracts revealed positive bioactivity results, and given that these are multi-component extracts, there is a potential for discovering multiple active compounds in a single fraction, making these organisms a valuable source of novel substances for future marine drug discovery. The very high antioxidant and antimicrobial activities observed could be associated with low-abundant substances, indicating a high potency for these compounds, especially for Bacteria 16 (*Shewanella* sp.), which revealed huge antioxidant and antimicrobial activities.

## 3. Experimental Section

### 3.1. Isolation and Purification of Epiphytic Marine Bacteria

Marine alga *Bifurcaria bifurcata* was collected from the Peniche coast and transported in seawater to the lab. Portions of alga were rinsed thoroughly with sterile seawater to remove loosely attached bacteria and then swabbed with a sterile cotton-tipped swab. The swab was then used to directly inoculate plates with Marine-Agar (peptone, 5 g; yeast extract, 1 g; FePO_4_, 0.1 g; agar, 15 g; dissolved in 1 L of seawater; pH 7.2–7.6), which were incubated at 20 °C. Colonies were selected at random and by systematic sampling, with distinctive colony types being selected along with a random sample from each plate. For purifying bacteria, we performed the streak technique at least 5 times.

### 3.2. Bacterial DNA Extraction and Identification

DNA was extracted from previously stored bacterial pellets with the GeneJET™ Genomic DNA Purification Kit (Thermo Fisher Scientific, Vilnius, Lithuania), according to the manufacturer’s instructions. Genus-level identification of isolates was determined by 16S rRNA gene sequencing. 16S rRNA gene fragments of *ca*. 1500 bp in length were amplified using the bacterial universal primers, F27 (5′-AGAGTTTGATCMTGGCTCAG-3′) and R1492 (5′-TACGGYTACCTTGTTACGACTT-3′) [16]. Reaction mixtures (25 mL) were prepared as follows: 8.75 µL of ultrapure water, 5 µL of GoTaq^®^ Flexi DNA Polymerase Buffer (Promega, Madison, WI, USA), 3 µL of MgCl_2_, 25 mM, 2.5 µL dNTPs, 2 mM, 1.5 µL of each primer, 10 µM, 1.25 of dimethyl sulfoxide (DMSO) 100%, 0.5 µL GoTaq^®^ Flexi DNA Polymerase 5U/µL and 1 µL of DNA template. Thermal cycling started with an initial denaturation step of 94 °C for 3 min, 35 cycles of 94 °C for 1 min, 55 °C for 1 min, 72 °C for 72 min and a final extension step of 72 °C for 10 min. All PCR amplifications were carried out in a Gradient Thermal cycler (Thermo Fisher Scientific, Waltham, MA, USA). All amplicons were checked under UV light after electrophoresis in 1.5% agarose gels stained with RedSafe™ (iNtRON Biotechnology, Seongnam-Si, Korea). PCR products with the right size (*ca*. 1500 bp) were cleaned with DNA Clean & Concentrator™-5 (Zymo Research Europe GmbH, Freiburg, Deutschland) columns and subjected to sequencing with the Sanger method in a BigDye Terminator 3 (Applied Biosystems, Foster City, CA, USA) using the forward and reverse primer. Nearly complete 16S rRNA gene sequences were obtained by sequencing with the forward and reverse primers. All sequences were automatically trimmed using the Phred quality score method [42]. Their closest phylogenetic relatives were searched for using the Basic Local Alignment Search Tool (BLAST, National Library of Medicine, Bethesda, MD, USA) of the National Center for Biotechnology Information (NCBI, National Library of Medicine, Bethesda, MD, USA) database.

### 3.3. Preparation of Epiphytic Bacteria Extracts

Isolated bacteria were cultured in 500 mL of Marine Broth (peptone, 5 g; yeast extract, 1 g; FePO_4_, 0.1 g; dissolved in 1 L seawater; pH 7.2–7.6) and incubated with aeration for three days at 25 °C. Bacteria cells were centrifuged and the pellet recovered and lyophilized. Lyophilized cells were then extracted with methanol and dichloromethane (1:1) at constant stirring for 12 h. The solvents were evaporated in a rotary evaporator at 40 °C, and the extracts were solubilized in dimethyl sulfoxide (DMSO) and stored at −20 °C until further use.

### 3.4. Biological Activities

#### 3.4.1. Antioxidant Activities

##### 3.4.1.1. Quantification of Total Phenolic Content (TPC)

The TPC of bacteria extracts was determined using the Folin-Ciocalteu method adapted to the microscale [43]. Briefly, 2 µL of sample were added to 158 µL of distilled water, 10 µL of Folin-Ciocalteu reagent and 30 µL of 20% sodium carbonate. After one hour of reaction in the dark, the absorbance was measured at 755 nm (Synergy H1 Multi-Mode Microplate Reader, BioTek^®^ Instruments, Winooski, Vermont, USA) and used to calculate the phenolic content using gallic acid as the standard.

##### 3.4.1.2. DPPH Radical Scavenging Activity

The DPPH free radical scavenging method was performed according to Brand-Williams (1995) [44] adapted to the microscale with slight modifications. The DPPH radical was dissolved in methanol (0.1 mM). Various concentrations of 2 µL of sample solution were added to 198 µL of the DPPH radical solution. The mixture was vortexed for 1 min and allowed to stand at room temperature in the dark for 30 min, at which time, the decrease in absorbance at 517 nm was measured (Synergy H1 Multi-Mode Microplate Reader, BioTek^®^ Instruments). The radical solution was freshly prepared each day. The synthetic antioxidant, BHT, was used as the standard. The ability to scavenge the DPPH radical was calculated using the follow equation:

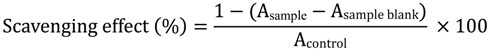
(1)
where A_control_ is the absorbance of the control (198 µL of DPPH solution plus 2 µL DMSO), A_sample_ is the absorbance of the test sample (198 µL of DPPH solution plus 2 µL sample) and A_sample blank_ is the absorbance of the sample in methanol (198 µL of MeOH plus 2 µL of sample).

##### 3.4.1.3. Oxygen Radical Absorbent Capacity (ORAC)

The oxygen radical absorbance capacity assay (ORAC) method was performed as described by Dávalos and co-workers [45] as follows: the reaction was carried out in 75 mM phosphate buffer (pH 7.4), and the final reaction mixture was 200 µL. The sample (20 µL) and fluorescein (120 µL; 70 nM, final concentration) were placed in the well of the microplate. The mixture was pre-incubated for 15 min at 37 °C. 2,2′-azobis (2-amidinopropane) dihydrochloride (AAPH), a free radical generator, was rapidly added using a multichannel pipet (60 µL; 12 mM, final concentration). The microplate was immediately placed in the reader and the fluorescence recorded every minute for 240 min. The microplate was automatically shaken prior to each reading. Trolox (6-hydroxy-2,5,7,8-tetramethylchroman-2-carboxylic acid), which is a hydrophilic analogue of vitamin E, was used to perform eight calibration solutions (1–8 µM, final concentration) on each assay together with a blank using phosphate buffer instead of the fluorescein.

Antioxidant curves (fluorescence *versus* time) were first normalized to the curve of the blank corresponding to the same assay by multiplying original data by the factor fluorescence_blank, *t*=0_/fluorescence_sample, *t*=0_. From the normalized curves, the area under the fluorescence decay curve (AUC) was calculated as:

AUC = 1 + *i* = 1*i* = 240*f*_i_/*f*_0_ (2)

where *f*_0_ is the initial fluorescence reading at 0 min and *f*_i_ is the fluorescence reading at time *i*. The net AUC corresponding to a sample was calculated by subtracting the AUC corresponding to the blank. Regression equations between net AUC and antioxidant concentration were calculated for all the samples. ORAC values were expressed as micromoles of Trolox equivalent/gram of extract by using the standard curve calculated for each assay.

#### 3.4.2. Antimicrobial Activity

The antimicrobial activity of bacteria extracts was evaluated against seven microorganisms: *Escherichia coli* (ATCC 25922), *Pseudomonas aeruginosa* (ATCC 27853), *Bacillus subtilis* (ATCC 6633) and *Salmonella enteritidis* (ATCC 13076) cultured in Luria broth (LB); *Staphylococcus aureus* (ATCC 25923) cultured in trypticase soy yeast extract medium (TSYE); and *Saccharomyces cerevisiae* (ATCC 9763) and *Candida albicans* (ATCC 10231) cultured in yeast extract peptone dextrose (YPD) medium. All mediums were obtained from Merck (Darmstadt, Germany).

Microorganisms growth was monitored spectrophotometrically (optical density at 600 nm (OD_600_)) during 48 h (data not shown) to determine the beginning of the exponential phase, in which the effect of bacterial extracts compared with the vehicle (microorganisms growth in the presence of 1% DMSO) will be assessed.

The antimicrobial assays were performed in 96-well plates, where 193 µL of medium, 5 µL of microorganism inoculum and 2 µL of extracts samples were added per well. Bacteria were incubated at 37 °C and fungi at 30 °C. As positive controls, ampicillin (0.01–1 µg/mL), amphotericin b (1–100 µg/mL), bacitracin (0.1–10 µg/mL), chloramphenicol (1–100 µg/mL) and oxytetracycline (0.01–1 µg/mL), purchased from Sigma Aldrich (Oakville, Canada), were used, previously prepared in sterile-filtered DMSO and stored at −20 °C. The ability of bacteria extracts to inhibit the microorganisms growth was accompanied by optical density at 600 nm (Synergy H1 Multi-Mode Microplate Reader, BioTek^®^ Instruments). Results were expressed in the percentage of control.

### 3.5. Statistical Analysis

Results are presented as the mean ± standard error of mean (SEM). The IC_50_ concentration was calculated from nonlinear regression analysis using the GraphPad Prism software with the equation: Y = 100/(1 + 10^(X − LogIC_50_)^).

## 4. Conclusions

To the best of our knowledge, for the first time, *Bifurcaria bifurcata*-associated bacteria were isolated and identified. This study reveals that *B. bifurcata* epiphytic bacteria are an excellent source of new natural antioxidant compounds, as well as antimicrobial compounds against Gram-positive bacteria. Moreover, all bacteria presented in this work are cultivable in a flask scale, which opens an opportunity for the production of the bioactive compounds in an industrial scale. As the production of secondary metabolites is related to environmental factors, such as UV radiation, pH, temperature, nutrient availability and time of fermentation, these can be manipulated in order to expose the bacteria to stress situations eliciting the production of the target bioactive compound. From a biotechnological perspective, the production of secondary metabolites, as effective competition and defense strategies by surface-associated microorganisms, constitutes an unparalleled reservoir for the discovery of new drugs, therapeutic agents and bioactive molecules, with applications across medical, industrial and environmental settings.
